# Parents' Views to Strengthen Partnerships in Newborn Intensive Care

**DOI:** 10.3389/fped.2021.721835

**Published:** 2021-09-27

**Authors:** Alexie Ferreira, Emanuela Ferretti, Krista Curtis, Cynthia Joly, Myuri Sivanthan, Nathalie Major, Thierry Daboval

**Affiliations:** ^1^Faculty of Medicine, University of Ottawa, Ottawa, ON, Canada; ^2^Department of Pediatrics, University of Ottawa, Children's Hospital of Eastern Ontario, Ottawa, ON, Canada; ^3^Neonatal Intensive Care Unit, Children's Hospital of Eastern Ontario, Ottawa, ON, Canada; ^4^Parents of Preemies Association, Ottawa, ON, Canada; ^5^Health Care Systems Division, Health Canada, Ottawa, ON, Canada; ^6^Telfer School of Management, University of Ottawa, Ottawa, ON, Canada

**Keywords:** NICU, parental involvement, partnership, family-centered care, newborn care, parental perspectives

## Abstract

**Background:** Parental involvement in their newborn's neonatal intensive care reduces stress and helps with the parent-child attachment, transition to home, and future development. However, parents' perspectives are not often sought or considered when adapting family-centered care in neonatal intensive care units (NICUs).

**Aim:** To identify what parents believe helps or hinders their involvement in their newborn's care when admitted to our Level 3B NICU.

**Methods:** Between August and October 2018, nine mothers and one father were interviewed during three 60- to 90-min audiotaped focus groups using a semi-structured interview tool. From the content analysis of the verbatims, three reviewers identified key themes that affected how involved parents could be in their newborn's care.

**Results:** Parents provided examples of factors that facilitated or restricted their involvement. The analysis identified themes: (1) parent-staff interactions, (2) supportive/trustworthy healthcare professionals, (3) consistency in care and caring staff, (4) family, couple, and peer support, (5) newborn status, (6) resources and education for parents, (7) the NICU environment, and (8) academic and research participation.

**Conclusion:** We identified a conceptual framework to allow our NICU team to prioritize working strategies to strengthen parental involvement in newborn care. In addition to implementing ways to involve parents, we need to address parents' satisfaction with their participation. These findings may help other investigators explore parents' expectations toward their NICU experience.

## Introduction

Recent advances in neonatal medicine and medical technology have made neonatal intensive care more accessible to fragile newborns, resulting in increased survival rates and lower limits of gestational age viability (1). But with all the monitoring, machines and equipment surrounding a newborn's fighting for survival, parents often feel lost and frightened and experience extreme stress and anxiety (2, 3), which can affect parent-infant attachments, hospital-to-home transition, and long-term development. Parental involvement with a newborn's day-to-day care in the neonatal intensive care unit (NICU) can reduce the anxieties, strengthen the bonds and ease the transition (4–6).

Family-centered care is a family-professional partnership developed to bring a more holistic and interdisciplinary approach to the care of newborns (3, 7). The newest models emphasize staff and family partnerships to better support infant and parent/infant outcomes (8). Shared governance allows for partnership with parents in co-designing a model of care that places the family unit at the center (9). Although principles of family-centered/integrated care models have been described (10), physical setup, workflow, culture characteristics, and staff experience vary considerably between NICUs (11). So each NICU must adapt their family-centered care models to fit parent's needs, considering the local context, culture, and staff experience, to successfully implement changes (11).

Although the Children's Hospital of Eastern Ontario's (CHEO) Level 3B regional NICU includes family-centered care practices, our internal patients' satisfaction surveys and unit performances to kangaroo care and other transitions, including discharge, showed challenges to parent participation in the care on their newborn during their NICU stay (12). From these observations, we identified the need to better incorporate parents' input to strengthen partnerships according to the model of shared governance (13). For this study, we explored parental perspectives to identify factors that help or hinder their involvement in their newborns' care. The research ethics boards of the CHEO Research Institute (CHEO REB# 18/33X) and the Ottawa Health Sciences Network (OHRI# 20180261-01H) approved the study.

## Materials and Methods

### Study Design and Setting

We adopted a qualitative descriptive phenomenological framework to explore how individuals interpret an experience (14) to better understand parents' participation in their newborn's care. The recommended guideline ‘Consolidated criteria for reporting qualitative research (COREQ): a 32-item checklist for interviews and focus groups' has been used to report this study (15). We conducted this study in a 16-bed surgical tertiary care Level 3B NICU. The clinical care team includes ~17 registered nurses, 10 respiratory therapists, one social worker, one dietician, one pharmacist, and 13 neonatologists.

### Participants

We used purposive sampling to recruit parents of high-risk neonates recently discharged from our NICU. The criteria to define high-risk neonates were: (1) infant born ≤29 weeks of gestational age; (2) infant with intraventricular hemorrhage grade III-IV, neonatal stroke and any other type of neonatal encephalopathy that present with seizures; (3) infant with a congenital anomaly in central nervous, respiratory, cardiac and/or digestive systems or genetic syndrome not yet identified who were hospitalized for at least 7 days, and/or (4) infant with hypoxic ischemic encephalopathy requiring therapeutic hypothermia hospitalized for at least 14 days.

The study design excluded any parents who could not speak English or any family in which parents present in the NICU were not deemed long-standing caregivers (e.g., adoption, foster care). Following pre-identification of the families according to the inclusion criteria and after being invited by the neonatal follow-up clinic nurse, our research assistant approached 21 parents for consent between June and September 2018. Although 15 parents initially consented, nine mothers and one father ultimately participated. The other five parents could not make it for scheduling conflict. Table 1 describes the demographic characteristics of their high-risk newborns, who were admitted between 2015 and 2018, and their parents.

**Table 1:** Demographic characteristics of high-risk newborns.

**Year of admission in the NICU**	**Gestational age (weeks + days)**	**Birth weight (g)**	**Gender**	**Pertinent neonatal complications**	**Developmental outcome**	**Parent**	**Parent age at birth**	**Parent education**	**Number of kids living at home**
2015	26 + 3/7	1,160	Male	Bilateral intraventricular hemorrhage grade 3; stable hydrocephalus; bronchopulmonary dysplasia	Normal	Mother	46	Unknown	2
2015	36 + 4/7	5,380	Male	Infant of diabetic mother; hypoxic-ischemic encephalopathy; hypothermia; neonatal seizures	Developmental delay with poor attention	Mother	40	Completed college or higher	3
2016	24 + 6/7	700	Male	Left intraventricular hemorrhage grade 4; bronchopulmonary dysplasia and home oxygenotherapy	Normal	Mother	26	Unknown	Unknown
2017	41 + 2/7	3,300	Male	Hypoxic-ischemic encephalopathy; hypothermia	Global developmental delay	Mother	23	Completed college or higher	1
2017	28+1/7	740	Male	Necrotizing enterocolitis; patent ductus arteriosus ligation; CoNS sepsis	Normal	Mother	33	Completed college or higher	1
2017	26 + 1/7	922	Male	Necrotizing enterocolitis stage 3 with bowel perforation; pulmonary valve stenosis; ventriculomegaly	Global developmental delay autism spectrum disorder	Mother	32	Unknown	2
2018	34+1/7	1,950	Male	Bilateral intraventricular hemorrhage grade 3; ventriculo-peritoneal shunt	Expressive language delay	Mother	20	Completed college or higher	1
2018	41 + 3/7	3,290	Male	Hypoxic-ischemic encephalopathy; hypothermia; neonatal seizures	Normal	Mother	29	Completed college or higher	1
						Father	Unknown	Completed college or higher	
2018	29 + 5/7	1,180	Male	Bilateral intraventricular hemorrhage grade 2; necrotizing enterocolitis; CoNS sepsis	Normal	Mother	32	Completed college or higher	2

### Data Collection

Between August and October 2018, three exploratory meetings with the parents—in groups of two, three, and five—were held at the hospital by one of the principal investigators, a physician with a master's degree (TD) and a researcher in healthcare system evaluation with doctorate degree (MM). Some parents might have interacted with one of the interviewers (TD) prior to the study, being a physician and medical director of the NICU. Interviewers used a semi-structured interview guide (Appendix 1) developed by two authors (TD, MM) who have substantial experience with qualitative research methods and interviews as well as extensive knowledge of NICU environment. We did not include an external review of the topic guide, considering the risk for bias as minimal. However, two principal investigators (TD, MM) revised the questions after each focus group to increase clarity and comprehensiveness and, to reflect the previous focus group's findings.

We asked the parents to identify and describe circumstances and conditions that facilitated or hindered their involvement in their newborn's care. We focused on specific events: medical rounds; critical situations; care of the newborn, including kangaroo care; breastfeeding; transition to home, and decision-making situations. Questions were open-ended and no examples were provided to parents, in order to explore their perspective without directing them toward a specific theme. Although small focus group facilitated symmetrical parents' participation, attention was given to silent parents to encourage them to share their opinion on each subject. To make parents feel more comfortable, they were given the opportunity to introduce themselves and their child at the beginning of the focus group. All the interviews were recorded and transcribed.

### Data Analysis

Two independent reviewers (AF, TD) used an inductive and constant comparisons process (16) to perform a qualitative content analysis of the first focus group interview involving five parents. One reviewer (AF) then conducted content analysis of the following two focus groups verbatim. Themes were designated as “facilitators” or “challenges” to parents' involvement. The two reviewers (AF, TD) met twice for a total of 4 h to review the coding and related transcripts and to discuss themes and concepts to create a framework. We counted the number of times parents mentioned examples to provide a relative weight between the themes. As a counter-coding strategy to ensure the validity of the results, the two coders met with a third author (EF) to review the themes and the preliminary classifications. Modifications were made as needed until agreement was reached. We used tables on Microsoft Word to support the analysis and organization of the data.

## Results

**Figure 1:**
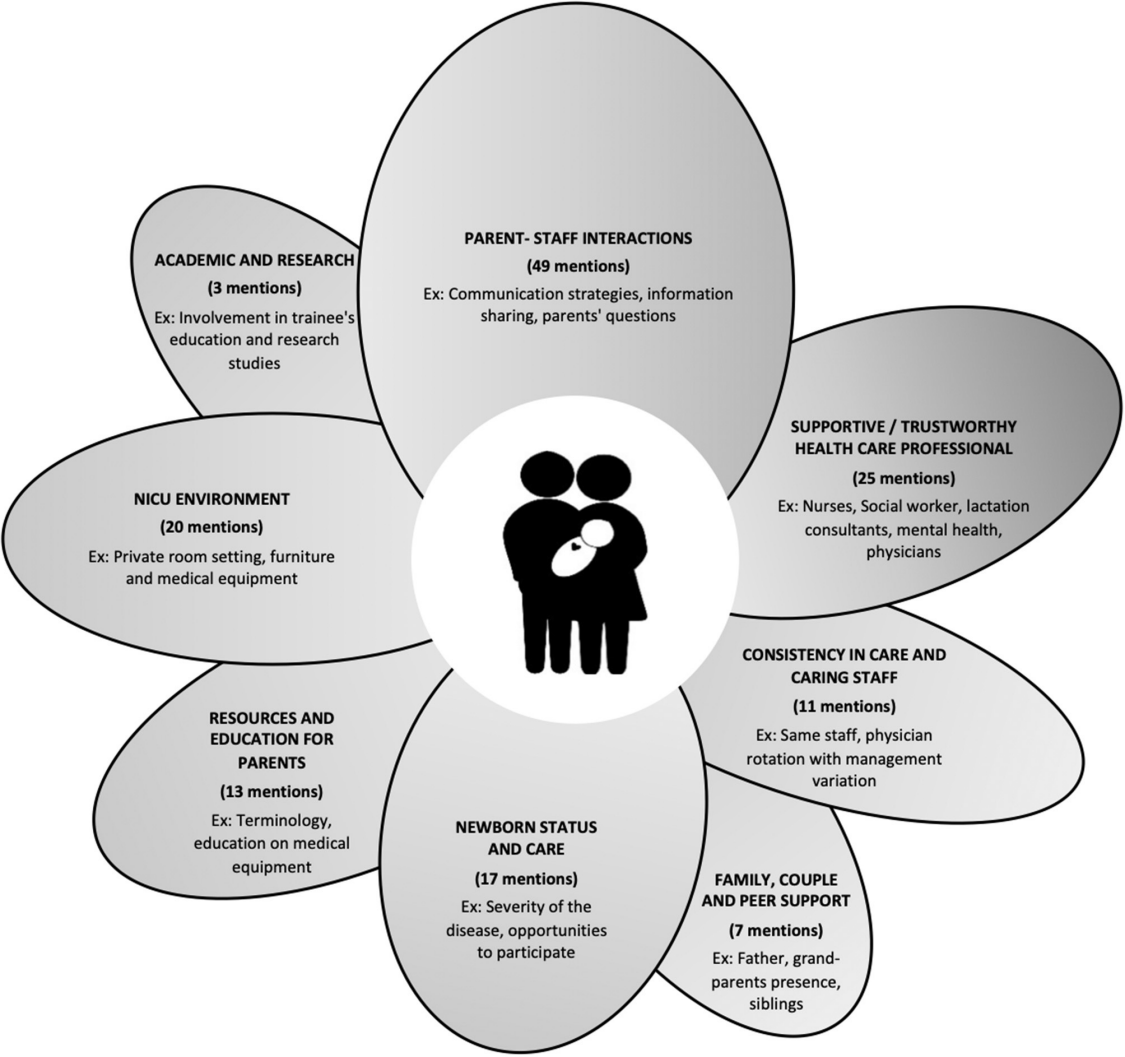
Themes of parental involvement in newborn care.

Eight themes were derived from the interviews, outlining factors that influenced parental involvement in their newborn's care during their experience in our NICU. These themes, outlined in Figure 1, include: parent-staff interactions; consistency in care and caring staff; supportive/trustworthy healthcare professional; newborn status and care; family, couple and peer support; resources and education for parents; NICU environment, and participation in academic curriculum and research studies. Tables 2–9 explain the themes and underlying sub-themes with paraphrased comments from the parents. Within the tables, we established each paraphrased comment as either a “facilitator” in white or a “challenge” in gray.

**Table 2:** Parent-staff interactions.

**Sub-themes**	**Paraphrastic coding**	**Situation**
Parents' questions	Less questions (especially during morning rounds) (FG#1)	Medical rounds
	Hard for parents to generate questions on the spot (FG#1)	
	‘Do you have any questions?' should be asked to parents at morning rounds (FG#3)	
	Feeling comfortable asking questions to better understand (FG#2)	No specific time or moment
Health care providers's attitudes	Parents felt included and respected when importance was given to their interpretation (FG#1)	Medical rounds
	Parents want to be acknowledged by the team at rounds (FG#3)	
	Rounds at bedside make parents feel like they are part of the discussion (FG#2,3)	
	Allowed to stay (choice given) and being explained: allows parents to see, instead of being reported second hand information (FG#1)	Critical situations
	Not rushed by staff to leave (FG#1)	In general
	Felt respected and included by doctors in the decision-making process (FG#1)	Decision making situations
	Parents are consulted for decisions (FG#1)	
	Can take time to think about the course of action before giving their decision (FG#1)	
	Not as much priority was put on family members (more on mother) (FG#1)	Kangaroo care
	Doctors do not seem to take time to “enjoy” the babies and know them more (FG#3)	In general
Communication strategies	Easier for parents to understand and follow the discussion if the staff starts with the head to toe, going system by system (FG#3)	Medical rounds
	To keep parents up to date, especially when morning rounds were missed (FG#1)	
	Parents would like a follow-up/recap session 1–2 h after morning round (post-medical round meeting) (FG#1)	
	Meeting with parents after rounds = Less questions (FG#1)	
	Good explanations given to parents one on one (FG#1,3)	Decision making situations
	They want to be asked “How involved do you want to be?” They want to do more (FG#1)	No specific time or moment
	Parents want to be called to be informed of procedures (ex: blood transfusion) (FG#3)	Decision making situations, Procedures
	Procedures are presented as options and requires consent (FG#1,2)	
Medical information sharing	Be careful about the wording and timing (FG#2)	Procedures
	Be careful with the words used and the amount of factual information given (FG#2)	Critical situations, Medical rounds
	Too much information at the same time: consent, explanations, etc., made parents feel overwhelmed (FG#1)	Critical situations, Procedures
	Different messages from different staff members made parents feel overwhelmed (FG#1,2)	No specific time or moment
	Hard for parents to understand everything (medical jargon) (FG#1)	Medical round
	Importance of medical terms (jargon), but there needs to be breaks to explain shortly to parents want is being said and make sure they understand (FG#3)	
	Lack of information and communication on the first days (FG#3)	Upon arrival at the unit
	Lack of information about kangaroo care. Need more information and to know when it is appropriate (FG#1,3)	Kangaroo care

^*^White color, Facilitator and Gray scale, Challenge.

**Table 3:** Supportive/trustworthy healthcare professional.

**Sub-themes**	**Paraphrastic coding**	**Situation**
Nurse	Parents know they will be called if anything happens and it made parents feel comfortable (FG#1)	Critical situations
	Nurse practitioner took time to explain and ask parents if they had questions (FG#3)	In general
	Lack of availability of experimented nurse (ex: for PICC line at night) (FG#2)	Night shifts procedures
	Parents felt free to ask questions and got answers from nurses (FG#1)	In general
	Updating parents on medical status, especially when morning rounds were missed (FG#1)	Medical rounds
	Parents can count on head nurse if there is an issue with a nurse (FG#1)	In general
	Reassuring comments: “not your fault” “an angel got me out of post-partum” (FG#1)	
Social worker	Would like an automatic appointment at the beginning (not just “as needed”) (FG#1)—“We have the social worker, the social worker comes by and says, “If you ever need to talk…” But I feel like I should have been appointed to her, like they should have just automatically make me do an appointment”	Upon arrival
	Provided money for gas and food, in order to help parents be present for their baby while feeling supported by the team (FG#1)	In general
	Good communication took time to listen to the mother and advocated for her (FG#3)	
	Presence at morning rounds is reassuring (FG#3)	Medical rounds
Lactation consultants	Available and helpful. Gives good comments/tips (FG#2)	Breastfeeding and feeding
	Should not shame parents or make them feel guilty (FG#3)	
Midwife	Good experience with staff (ex: midwife's support with breastfeeding) (FG#1)	
Mental health	Need of psychologists or psychiatrists to check on the mother after the “traumatic” experience (FG#3)	Upon arrival at the unit, In general
Physician	If needed, they know the doctor will take a good decision for them (trust in the doctor) (FG#1)	Decision making situations
	It gives motivation to have a tangible plan (to breastfeed for example) (FG#1)	Breastfeeding and feeding
	More acknowledgment or consideration from doctors for parents' opinions is needed (FG#3)	Medical rounds, Decision making situations

^*^White color, Facilitator.

^*^Gray scale, Challenge.

**Table 4:** Consistency in care and caring staff.

**Sub-themes**	**Paraphrastic coding**	**Situation**
Physician rotation on the week-end and every 2 weeks	Felt that there was no progress (FG#1)	Plan of care
	Change of plan: barrier to participation (FG#1)	
	Accepted by parents (FG#1)	In general
	Doctors rotation is a barrier to consistency of management plan because they have different opinions (FG#3)	Plan of care
Same staff team	Feeling of connection (trust) (FG#1)	In general
	No need to repeat everything (FG#1)	
	Facilitate participation (FG#1)	
	Enhance family's comfort level (FG#1)	
	Following a change of nurse, parents felt less comfortable because the nurse was usually less permissive with them (FG#1)	
Interdisciplinary	Collaboration between doctors, dieticians and parents (FG#2)	Plan of care, Decision making situations

^*^White color, Facilitator.

^*^Gray scale, Challenge.

**Table 5:** Family, couple and peer support.

**Sub-themes**	**Paraphrastic coding**	**Situation**
Father's role/support	Possibility for father to help feeding and milk the baby (FG#1,2)	Breastfeeding and feeding
Extended family/grandparents	Grandparents' presence to support parents in difficult moments (FG#1)	Difficult moments
Couple	Making sure parents are doing fine as a couple and as a family (FG#3)	In general
Siblings	Hard to take care of other kids while visiting NICU and staying at the hospital (FG#3)	Outside of NICU
Peer support	Assigning a new mom in the NICU with a mom who's been through it (FG#3)	In general

^*^White color, Facilitator.

^*^Gray scale, Challenge.

**Table 6:** Newborn status and care.

**Sub-themes**	**Paraphrastic coding**	**Situation**
Medical procedures/interventions	In need of the smaller number of attempts possible (FG#2)	Critical situations, Procedures
	Not do them in front of parents (would make them panic) (FG#2)	
Opportunities to give care to their baby	Less motivated when feeding tube or difficulties with breastfeeding (FG#1)	Breastfeeding and feeding
	Importance of production of good milk supply (FG#1)	
	Breastfeeding to bound with baby (FG#1,2)	
	Importance of skin-to-skin contact. Really emotional and allows parents to create a strong bond with the baby (FG#1,2)	Kangaroo care
	Importance of having more opportunities for kangaroo care (FG#1)	
	It is important to be there for the first bath of the baby (FG#3)	First moments
	To see their baby especially the first time after giving birth (FG#2)	
	Impossible to touch the baby (FG#1)	
Severity of newborn's disease	Mother was detaching from the baby (FG#1)	Critical situations

^*^White color, Facilitator.

^*^Gray scale, Challenge.

**Table 7:** Resources and education for parents.

**Sub-themes**	**Paraphrastic coding**	**Situation**
Terminology	Would like a course or access to resources for terminology (FG#1)	Upon arrival at the unit/Medical rounds
“Welcomer” or info session	Could greet the parents on their first time on the floor and give them information (FG#3)	Upon arrival at the unit
Education on equipment/medication and medical care	Parents learned how to remove monitor when needed (FG#1)	Discharge and transition to home, all care or contact with the baby
	Parents would like to help and be taught about the medical equipment (FG#1)	
	Bedside teaching (monitors, etc) gives parents confidence, independency and understanding (FG#1)	
	More hands-on required before discharge (oxygen tank) and dry-run (ex: how to wrap the baby) (FG#1)	
	Good teaching on medications (FG#1)	
	Good preparation and hints from nurse to gradually gain independency and confidence (FG#2)	

^*^White color, Facilitator.

^*^Gray scale, Challenge.

**Table 8:** NICU environment.

**Sub-themes**	**Paraphrastic coding**	**Situation**
Equipment vs. Breastfeeding	Importance of having access to a pump and to breastfeed beside the incubator (instead of pump room) (FG#1)	Breastfeeding and feeding
	Would like breastfeeding pillows “u-shaped” (FG#2)	
Furniture	Uncomfortable chairs (FG#2)	In general, all care or contact with the baby
	Prefer chairs that can be extended to become beds (FG#2)	
	Would like a double bed in the room to stay with partner and baby, and share emotions (FG#1)	
Medical equipment	Medical equipment (tubes, CPAP, wires, machines beeping) (FG#2)	
	Intimidating, barrier to kangaroo care (FG#1)	
White board	Connection and communication tool (FG#2)	In general
	Needs to be promoted and explained to parents to make them feel comfortable using the white board (FG#3)	
Privacy	Room's curtains, always open, but closed when privacy needed (ex: breastfeeding) (FG#1)	In general, all care or contact with the baby
	Pods—Room Felt like an apartment (comfortable) (FG#1)	
	Important to have privacy during rounds (CHEO vs. TOH) and not being exposed to other parents (FG#2)	In general, Medical rounds
Crowdedness	Many people working around (FG#1)	In general
NICU opened 24/7	Would like to be told it is 24/7 (FG#1)	From early admission
Resting out of NICU moments	Allows them to go home and rest (FG#1)	Outside of NICU
Feeling connected/Call-in	Able to take a break, can call in at anytime (FG#1)	

^*^White color, Facilitator.

^*^Gray scale, Challenge.

**Table 9:** Academic and research participation.

**Sub-themes**	**Paraphrastic coding**	**Situation**
Trainees/students	Importance of letting students learn and have hands-on experiences (FG#1)	In general, Procedures
Research	Importance of parent's involvement in research projects (FG#2)	In general

^*^White color, Facilitator.

During the interviews and when reading the verbatim, it became obvious that parents weighted some elements more than others. Figure 1 describes how many times each theme was mentioned by parents during the interviews. For example, “parents-staff interactions” was clearly an important element, being mentioned 49 times by parents, while “academic and research participation” was only mentioned three times.

### Parent-Staff Interactions (Table 2: 49 Mentions)

Parent-staff interactions clearly influence parental involvement in the NICU care of their newborns. Parents noted the importance of the interactions and communication strategies with the nurses, physicians and social workers, saying they should be considered in daily care, acknowledged by the team and provided time to think when a course of action has to be decided. Morning rounds are a crucial moment for parents as they receive updates about their newborn and an opportunity to ask questions.

“*It's very easy if they talked at the bedside of the baby, then you're obviously part of the conversation, and they always asked me at the end of their discussion, they asked me if I had any questions and concerns.” (FG#2)*

This demonstrates the importance of staff and physicians' attitudes, the type of communication strategies used, and the need for sharing medical information, allowing questions and asking for consent about the procedures used on the newborns.

### Supportive/Trustworthy Healthcare Professional (Table 3: 25 Mentions)

Parents' description of what influenced their participation in their infant's care reflected staff attitudes to making them feel comfortable.

“*One nurse, she snapped me out of my postpartum. Just as — you know, no matter what anybody was saying to me, it was just — it was constant, I was blaming myself. But her, she was like — to me, she was like my angel. She came in, she's like, “No, that's enough. You're doing perfect, that's more than enough.” (FG#1)*

Nurses' availability, openness to questions and experience influenced parents' involvement as they felt comfortable asking questions. They could rely on nurses for explanations, updated information and reassurance. In addition, parents identified the lack of experienced nurses on night shifts as a barrier to support.

Parents trusted physicians in making good decisions during critical situations. On the other hand, they would appreciate more consideration from physicians for parents' opinions. “*After I got into an argument with one of the doctors. After I had told him like, ‘No, I do not want Puramino or lipid or formula.' He's like, ‘Okay, fine. Then what's going to happen after this then?' I'm like, ‘I don't know what's going to happen after this. Right now, I don't want him to get NEC again.' for, like, the fifth time.” (FG#3)*

Social workers were identified as valued in many ways, from helping understand the financial aspects of having a preemie to being present during morning rounds. The social worker was felt to be reassuring, enabling good communication and providing advocacy.

“*So I found CHEOs family-centered approach, and the […] social worker was making sure we were okay as a couple and as a family. That made us stronger than ever.” (FG#3*)

Parents said they would appreciate an automatic appointment with a social worker at the beginning of their stay instead of only if needed.

Lactation support was also seen as beneficial. One mother mentioned that additional access to mental health support from a psychologist or psychiatrist would have been helpful to cope with this traumatic experience.

### Consistency in Care and Caring Staff (Table 4: 11 Mentions)

Consistency in care is an important concept for the parents. In fact, parents preferred to have the same staff to better participate in the care and be comfortable with and trust the care team. The physician rotation on weekends and after every 2 weeks was perceived as a barrier to the parents' involvement. They noted little to no progress over the weekend and perceived significative changes in care plans with physician rotation, which made their involvement more difficult.

“*If the same physician was in for two weeks, you get momentum going and you would kind of be — you'd have a plan, more of a long-term plan. When someone would come in on the weekend, sometimes they would totally switch gears on you. And then, whoever was there would come back the next week, it would sometimes shift again.” (FG#3)*

### Family, Couple, and Peer Support (Table 5: 7 Mentions)

The presence of family members is significant. The parents noted the importance of their own wellness and indicated the difficulty in caring for the newborn's siblings as well as visiting the NICU, resulting in pressures that can damage the couple's relationship.

“*When your husband and yourself […] are under a time of stress, you can say the nastiest things to each other, and relationships can falter away.” (FG#3)*

Family members' presence can play a crucial role in supporting the parents' involvement with the newborn's care.

They also mentioned the need for peer support and the opportunity of a new mother to be paired with another parent whose newborn was admitted to the NICU.

“*Peer support, so just like assigning one mom with, like, a new mom in the NICU with a mom who's been through it.” (FG#3)*

### Newborn Status and Care (Table 6: 17 Mentions)

Parents want to be taught about various care procedures so they can gain independence and confidence leading to the infant's eventual discharge. Providing opportunities to breastfeed and have skin-to-skin contact could improve parental involvement if associated with adequate teaching support at the bedside. Skin-to-skin contact is “*[…] very important. It just encourages the mother to attach.” (FG#2)*

### Resources and Education for Parents (Table 7: 13 Mentions)

Parents would benefit from a variety of targeted resources. For example, they said a course or resources for medical terminology, basics of the NICU, and an informative session on their first day at the NICU would be helpful.

“*There should be some sort of way to present to parents, this is the NICU, this is what you can expect kind of thing, like, just one information session. Parents should be invited to one information session.” (FG#3)*

Education about the medical equipment (e.g., monitors, nasogastric tubes, etc.) and medications should also be offered to build their confidence. Parents wished for more hands-on experiences to gain confidence in caring for their newborn. It allows parents to feel closer to their newborn and facilitates their discharge from the unit because they feel more ready.

### NICU Environment (Table 8: 20 Mentions)

Parents appreciated comfortable chairs that extend to become beds and, as privacy is an important factor, curtains and a quieter environment. They suggested breastfeeding could be improved by u-shaped pillows and good accessibility to breastfeeding pumps beside the incubator. Parents need to be comfortable in order to better participate in the care.

“*The chairs are horrible, and the nurses were trying to put a pillow and make me comfortable. […] So as much as I wanted to stay there all day long, I was not comfortable.” (FG#2)*

White boards in the rooms where parents and staff can exchange messages facilitate communication and are appreciated as a connection tool but need to be promoted and explained to make the parents feel comfortable using them.

They appreciated that they could call the unit at any time, saying it let them take breaks when needed, or to go home and rest, and still feel connected to their newborns at all times.

### Academic and Research Participation (Table 9: 3 Mentions)

Although less often mentioned, some parents brought up the importance of being able to decide whether to be involved in research projects, with some saying it is gratifying to participate in the development of the next generation of treatments to help future families and fragile newborns. They also appreciated the chance to participate in the education of future staff by allowing students to have hands-on experiences. It gave parents the sense of being “*part of the medical team or part of the healthcare team.” (FG#2)*

## Discussion

From parents' testimony about their involvement in the care of newborns admitted in the NICU, we identified eight themes that influenced their satisfaction. Parent-staff interactions were significant in developing trust. Examples of the situations discussed range from admission to the NICU, medical rounds, breastfeeding, kangaroo care, decision-making processes and transition to home. The wide variation of examples given by parents demonstrates the need for flexibility when implementing a framework within an NICU to facilitate parents' involvement in the care of their newborn.

Research on family-centered care on pediatric wards happened well before Harrison published, in 1993, principles for NICUs. Back then, allowing parents to have a say, negotiating with them, involving them in decision-making and overall parent involvement was perceived as complex (17). There is still resistance from NICU staff about parents' participation in the decision-making process and care of the newborns (18) but we believe use of the themes identified in our study to educate our staff will help alleviate resistance. Studies have shown that the resistance may be related to the lack of knowledge about parents' perceptions about their involvement in newborn care (19).

The importance for the healthcare professional to understand and believe in the philosophy of improving parental participation is not new. Early in the 1990s, Palmer et al. reported that the success of parental involvement in their child's care was conditional on the parents' and staff's attitudes and willingness to work together (19). The authors described the importance of good understanding of parental role in the hospital and the importance of the emotional support to families. Although their recommendations were aimed at pediatric wards, the results of our study would support similar approaches for NICUs. For example, the stress on a couple of having a newborn in intensive care can result in lack of intimacy and communication, depression, anxiety and increased risk of marital dissolution (20). The team should acknowledge possible adjustments needed for the couple to help in the care of their newborn. Peer-to-peer support was also mentioned by our participants. This support program foster feelings of safety and comfort (21) and encourage them to better advocate for their newborns (22).

More than 25 years ago, Harrison stated the importance of an open and honest communication between parents and professionals (17). Our findings on the importance of the parent-staff interactions are aligned with this description. Lopez-Maestro et al. (6) described a framework to improve parental involvement and parent-infant interactions, called “infant and family-centered developmental care.” It identified seven themes: 24-h parental access; psychological support for parents; pain prevention, assessment and treatment; supportive environment; postural support; skin-to-skin contact; sleep protection, and breastfeeding and lactation support. Although our classifications are different, we found many similarities with their findings. The difference is explained in part by the higher level of categorizations in our study. One of the major differences we found is the prime importance of parent-staff interactions. Enabling parental participation is largely influenced by the nurse acknowledgment of the parents as the main caregiver and awareness of the benefits of family-centered care philosophy and skin-to-skin care (11). The healthcare team's attitude may be influenced by unit policies about inviting parents to medical rounds (23). Our category about availability and supportive staff reflects that important factor. Family-centered care should be viewed as a philosophy aiming to improve parental participation (24, 25). The acknowledgment of this view is a first step to modifying parent-staff interactions to positively influence parents' involvement in the care of newborns. Although themes could have been amalgamated, such as “parents-staff interaction” and “Supportive/trustworthy healthcare professional,” we identified these themes based on their pertinence for elements to guide future improvements in developing our family-centered care model.

Some NICU family-centered care approaches are developed from healthcare professionals' opinion agreed or adapted by selected parents (10). Such models could have been implemented in our unit, but some of the proposed strategies—such as giving report, basic charting and maintaining a diary or providing focused medical care—are professionally goal-oriented and may not be appropriate for all parents (10). More recently, Skene et al. demonstrated the importance of understanding the unique context of the NICU to support cultural changes (11). In accordance with our results, they identified similar areas of changes, including providing family support and educational programs.

Our study on the perspective of the parents will support change in the philosophy and creation of educational programs to improve attitudes toward parents' participation in newborn care. We will use the examples brought by the parents to guide discussions during interviews with NICU staff. By exploring parental testimony and identifying strategies, we will facilitate parental participation in the care of newborns in different situations.

As Skene et al. mentioned, we needed to better understand the unique context of our NICU before discussing family-centered care with our staff (11). Although some of the themes we found are potentially specific to our unique environment, the higher level of abstraction of our findings into the themes identified is broad enough to be adapted to other NICUs. In addition, the situations identified by parents—early admission in the NICU, medical rounds, critical situations, decisions making and planning of care, breastfeeding, skin-to-skin, kangaroo care to transition to home—are similar between NICUs, which could facilitate the use of our findings for their benefit (26).

Although the impact of parents' participation in the care of their newborn was not our primary goal, the parents explained many potential benefits, including improving milk production, bonding with the newborn, and developing a sense of competence and confidence. These complementary findings are supported by the literature (27).

The results of our study were discussed by our Family Partnership in NICU Care advisory committee and, in 2019, up to three parents were included to help design approaches to improve parental participation in newborn care.

### Study Limitations

Although the sample size was small, at 10 participants, the qualitative design of the study allowed in-depth exploration of parents' experience that covered a range of perspectives, echoing the findings of Russell and Gregory in 2003 (28). The participation of parents who experienced the life in the NICU over a large period, 2015 to 2018, and parents' detailed description of their experiences obtained after three focus groups, provided a good understanding of the themes that influenced their participation in the care of their newborn, using the concepts of saturation outlined by Hennink et al. in 2019 (29). The focus groups were held in English, which may have been a language barrier for parents from different origins or cultures. Understanding of cultural impact is limited by the small number of parents, which did not include the indigenous population among the parents. The participation of only one father precludes any conclusion based on gender. Although similarities in parenting role exist among diverse cultures and religions, the concept of “care” might vary (30). The influence of parental gender, ethnicity, culture and beliefs will need to be explored in future studies.

The focus groups were led by a lead consultant on the ward, who may have previously interacted with parents. Therefore, a potential bias exists if parents felt unable to sincerely answer questions or express their perceptions and concerns. The interview guide not being externally reviewed could be considered a limitation, however strong experience of the interviewers with the environment and specific attention to use open-ended questions without suggestive examples or answer choices mitigated this risk. The analysis process maybe criticized, but the involvement of a third coder as a counter-coding strategy alleviates this caveat (28). Participants did not provide feedback on the transcripts or on the findings as our methodology did not include a triangulation process. Follow-up with the parents to confirm whether our findings reflected their experiences or if parents had new information to add was not possible. Despite the limitations, we believe that the themes identified may be of use to NICU leaders exploring their own environments.

## Conclusion

This study provided eight themes in which a wide variety of examples could be used to guide improvements for parental involvement in the care of their newborn in our NICU. As such, a personalized approach focused on each parent's specific needs is an innovative approach to enhance care delivery. Parents' insight should facilitate practice improvements. To implement new approaches that will include these themes, the next step would be to investigate the perspective of healthcare professionals in light of our findings. The goal would be to strengthen partnership between healthcare professional and families to provide individualized support to families of newborns admitted in the NICU.

## Data Availability Statement

The raw data supporting the conclusions of this article will be made available by the authors, without undue reservation.

## Ethics Statement

The studies involving human participants were reviewed and approved by Children's Hospital of Eastern Ontario Research Institute Research Ethics Board. The patients/participants provided their written informed consent to participate in this study.

## Author Contributions

AF, TD, and MS conceptualized and designed the study with significant contributions of EF, KC, CJ, and NM. AF, TD, and EF performed the analysis. KC, CJ, MS, and NM reviewed the analysis for final approval. AF wrote the first draft of the manuscript under the supervision of TD. EF, KC, CJ, MS, and NM reviewed the manuscript, figures, and tables. All authors contributed to manuscript revision, read, and approved the submitted version.

## Conflict of Interest

The authors declare that the research was conducted in the absence of any commercial or financial relationships that could be construed as a potential conflict of interest.

## Publisher's Note

All claims expressed in this article are solely those of the authors and do not necessarily represent those of their affiliated organizations, or those of the publisher, the editors and the reviewers. Any product that may be evaluated in this article, or claim that may be made by its manufacturer, is not guaranteed or endorsed by the publisher.

## Supplementary Material

Appendix 1 Exploratory Focus groups with parents—semi-structured interview guide.
