# Supplementary material for: Parents' Views to Strengthen Partnerships in Newborn Intensive Care
Source: Front Pediatr. 2021 Sep 27;9:721835. doi: 10.3389/fped.2021.721835 (PMC8504452; doi:10.3389/fped.2021.721835)
Supplement: Appendix 1 — Exploratory Focus groups with parents—semi-structured interview guide. [file Data_Sheet_1.pdf]

Appendix 1. Exploratory Focus groups with parents - semi-structured interview guide

After a short introduction of the interviewer and each participant, questions to be used by the research assistant during the interviews.

1) Can you describe how you would prefer to participate in the care of your newborn?

2) What within the NICU workflow or day-to-day experience …

   a. … helped or would have helped you better understand your newborn needs so you can help with his care?

   b. … helped or would have helped you in the transition between the NICU and home?

   c. … should be changed or improved for you to facilitate your involvement in the day-to-day care of your baby?

3) What education do you think would help you better understand your baby's needs or would help you feel more comfortable participating in the care of your newborn?

4) What challenges did you experience when your baby was discharged from NICU?

   a. What could have helped you?

5) What challenges did you experience in the first few weeks after your baby was discharged from home

   a. What could have helped you?

As per semi-structured interview methods, additional questions will be prompted by the participants' responses to describe better and understand parents' perspectives and educational needs.
